# The impact of insurance status on patient placement into inpatient and outpatient orthopaedic surgical centers

**DOI:** 10.1186/s13018-024-04734-8

**Published:** 2024-06-28

**Authors:** Jetha Tallapaneni, Michael Harrington, Zach Troiani, Luciano Venturino, Andrew Rosenbaum

**Affiliations:** Department of Orthopedic Surgery, Albany Medical Center, Albany, USA

**Keywords:** Total joint arthroplasty, Practice management, Insurance status, Quality of care, Outpatient, Arthroplasty

## Abstract

**Background:**

Innovation has fueled the shift from inpatient to outpatient care for orthopaedic joint arthroplasty. Given this transformation, it becomes imperative to understand what factors help assign care-settings to specific patients for the same procedure. While the comorbidities suffered by patients are important considerations, recent research may point to a more complex determination. Differences in reimbursement structures and patient characteristics across various insurance statuses could potentially influence these decisions.

**Methods:**

Retrospective binary logistic and ordinary least square (OLS) regression analyses were employed on de-identified inpatient and outpatient orthopaedic arthroplasty data from Albany Medical Center from 2018 to 2022. Data elements included surgical setting (inpatient vs. outpatient), covariates (age, sex, race, obesity, smoking status), Elixhauser comorbidity indices, and insurance status.

**Results:**

Patients insured by Medicare were significantly more likely to be placed in inpatient care-settings for total hip, knee, and ankle arthroplasty when compared to their privately insured counterparts even after Centers for Medicare and Medicaid Services (CMS) removed each individual surgery from its inpatient-only-list (1.65 (*p* < 0.05), 1.27 (*p* < 0.05), and 12.93 (*p* < 0.05) times more likely respectively). When compared to patients insured by the other payers, Medicare patients did not have the most comorbidities (*p* < 0.05).

**Conclusions:**

Medicare patients were more likely to be placed in inpatient care-settings for hip, knee, and ankle arthroplasty. However, Medicaid patients were shown to have the most comorbidities. It is of value to note Medicare patients billed for outpatient services experience higher coinsurance rates.

**Level of evidence:**

III.

## Introduction

Due to advances in technology and increasing demand, arthroplasty is increasingly viewed as an outpatient procedure [1, 2]. Specifically, demand for total knee arthroplasty (TKA), total shoulder arthroplasty (TSA), total ankle arthroplasty (TAA), and total hip arthroplasty (THA) has increased dramatically. Surgeries performed in outpatient centers are not only more cost-effective, but also convenient for the physician and the patient, reducing time spent in the hospital and allowing for more home-recovery options [1]. Multiple studies have displayed that with carefully selected patients, outpatient arthroplasties were equivalent, with similar short-term complications, revision rates, decreased costs, and comparable readmissions [3–7]. The efficacy of outpatient arthroplasty in appropriately selected patients has been empirically demonstrated.

Though many studies have attempted to compare surgical costs and outcomes between patients treated in outpatient and inpatient centers, few have attempted to explore the reasons why patients were assigned to different care settings for the same treatment [7, 8]. The purpose of this study, therefore, was to determine what factors other than co-morbidities were implicated in assigning certain patients to either an inpatient or outpatient care setting for procedures removed from the CMS inpatient only list. Specifically, this study seeks to determine if insurance/primary payer status (Medicare, Medicaid, private insurance, uninsured, dual coverage) or other patient characteristics have an impact on patient placement into either an outpatient or inpatient care center for arthroplasty.

**Table 1:** Patient Characteristics as Stratified by Insurance Status

Insurance Status	Private	Medicare	Medicare Managed	Medicaid	Medicaid Managed	Other
Number of Patients	2,424 (35.2)	1,954 (28.3)	1,937 (28.2)	15 (0.22)	270 (3.9)	282 (4.1)
**Surgical Time (mins)**	130.8 ± 33.4	132.3 ± 42.9	129.0 ± 30.8	142.0 ± 37.3	138.5 ± 52.2	141.9 ± 41.6
**Age (yrs)**	58.5 ± 8.2	72.2 ± 8.5	72.6 ± 7.2	57.2 ± 10.6	54.3 ± 10.8	58.3 ± 9.8
**Race**						
Caucasian	2,249 (35.7)	1,823 (28.9)	1,786 (28.3)	9 (0.14)	193 (3.0)	245 (3.9)
African American	78 (29.9)	48 (18.4)	64 (24.5)	1 (0.4)	43 (16.5)	27 (10.3)
Asian	10 (24.4)	11 (26.8)	11 (26.8)	0**^** (0)	8 (19.5)	1 (2.4)
Other	83 (32.0)	67 (25.9)	71 (27.4)	4**^** (1.5)	26 (10.0)	8 (3.1)
**Care Setting (%)**						
Inpatient	56.6	66.9	59.4	60	49.3	61.3
Outpatient	43.4	33.1	40.6	40	50.7	38.7

Numbers in parentheses are percentages

**^** Numbers were significant to *p* < 0.05 with Fisher’s Exact Analysis

## Methods

De-identified data from patients who underwent both inpatient and outpatient primary TKAs, THAs, TSAs, and TAAs was collected from a single, tertiary-care academic medical center and affiliated outpatient facilities in Upstate New York. The population served by these facilities is socioeconomically diverse, providing the presence of various insurance/payer models (Table 1). A total of 6,882 patients were included in the study, of which 3,456 underwent THA, 2,851 underwent TKA, 417 underwent TSA, and 158 underwent TAA (Table 2). Of the patients studied, 2,738 underwent outpatient surgery (39.7%). The only inclusion criteria was that patients must have undergone arthroplasty between 2018 and 2022. Of the 6,882 patients, 15 (0.002%) had expired during the hospital visit in which the arthroplasty was performed–these patients were excluded from the study. Outpatient surgery was done at either one of two outpatient centers or at the academic medical center as an outpatient—all outpatients were discharged on the same day. Inpatient surgery was done at the academic medical center and patients spent at least one night at the hospital. Data regarding surgical setting (inpatient vs. outpatient), covariates (age, gender, race, obesity, smoking status), Elixhauser index scores, and insurance status (primary payer status: Medicare, Medicaid, private insurance, uninsured, dual coverage) was obtained. This project was exempt from the IRB as determined by Albany Medical College.

**Table 2:** Summary Statistics of Patient Population, Stratified by Type of Arthroplasty

	THA (50.2%) 3,456	TKA (41.4%) 2,851	TSA (6.1%) 417	TAA (2.3%) 158
**Inpatient Cases**	2,372 (68.6)	1,462 (51.3)	239 (57.3)	71 (44.9)
**Outpatient Cases**	1,084 (31.4)	1,389 (48.7)	178 (42.7)	87 (55.1)
**Age**				
<65	1474 (46.0)	1105 (43.2)	153 (39.3)	86**^ (**57.6)
>65	1866 (54.0)	1620 (56.8)	253 (60.7)	62**^** (42.4)
**Race**				
Caucasian	3173 (50.3)	2609 (41.4)	381 (6.0)	142 (2.3)
African American	127 (48.7)	114 (43.7)	14 (5.4)	6 (2.3)
Asian	17 (41.5)	24 (58.5)	0 (0)	0 (0)
Other	127 (49.0)	101 (39.0)	22 (8.5)	9 (3.5)
**Gender**				
**Men**	1619 (46.8)	1275 (44.7)	221 (52.3)	90 (56.9)
**Women**	1837 (53.2)	1576 (55.3)	196 (47.7)	68 (43.1)
**Insurance**				
Private	1,280 (37.0)	971 (34.1)	107 (25.7)	66 (41.8)
Medicare/Managed	1934 (55.9)	1619 (56.8)	260 (62.4)	78 (49.4)
Medicaid/Managed	151 (4.4)	115 (4.0)	14 (3.4)	5 (3.2)
Other	91 (2.6)	146 (5.1)	36 (8.3)	9 (5.7)
**Co-Morbidity Index**				
Elixhauser	3.33	1.39	2.13	1.58

Numbers in parentheses are percentages

^ Numbers were significant to *p* < 0.05 with t-test analysis

Comorbidities were given special attention as previous studies displayed the key role co-morbid disease may play in the care-setting decision. Two indices, the Charlson-Deyo and the Elixhauser were considered. Both have their benefits. Recent studies have advocated for the Elixhauser as it considers more co-morbidities, potentially allowing better discrimination when compared to the Charlson-Deyo [9, 10]. Given this new research, the Elixhauser score was chosen for analysis [10].

Patients were stratified by type of arthroplasty performed. Within each of these arthroplasty groups, there were two subgroups consisting of the two care settings considered: hospital inpatient and outpatient units. Within each of these subgroups, patients were further classified into six categories based on their insurance status. The insurance statuses considered included: Medicare, Medicare Managed, Medicaid, Medicaid Managed, private insurance, and “other” (consisting of uninsured, self-pay, law enforcement). Since all patients treated fell into one of these six categories, each subgroup would consist of a varying percentage of each of these categories.

### Statistical analysis

Ordinary least squares (OLS) regression analysis was used to determine how insurance status and patient age were associated with the Elixhauser index score. Binary logistic (BL) regression was used to determine the odds (OR) that a patient with a certain insurance status was assigned to one care setting over another for a particular procedure, given all other covariates considered, after the date that particular procedure was removed from CMS’s inpatient only list (January 2018 onwards for total knee arthroplasty, January 2020 onwards for total hip arthroplasty, and January 2021 onwards for total ankle and shoulder arthroplasty) [11].

The primary variable assessed through the BL regression equation was choice of care-setting and its relationship to the Elixhauser co-morbidity index values, age, and gender. The relationship between these variables was further stratified by insurance status and surgery type. This level of analysis allowed this study to draw conclusions regarding the impact Elixhauser index scores and insurance status had on patient placement into either care setting. This method of analysis has been employed by studies in the past that sought to analyze data in a similar fashion and has been proven to be effective [12]. Independent t-tests and Fisher’s exact tests (*p* < 0.05) were used to determine if there were any significant differences found between the patients undergoing the individual types of surgery analyzed as well as between patients insured by each of the payors considered. The variables analyzed with the t-test included age and surgical time. The variable analyzed with the Fisher’s exact test was racial composition.

**Table 3:** Association between Elixhauser Score and Relevant Variables- OLS Regression Output

	Coefficient	Confidence Interval	P-Value
**Admit Age**	0.135	0.119–0.152	< 0.01^
**Medicare**	1.560	1.155–1.965	< 0.01^
**Medicare Managed**	0.613	0.199–1.026	< 0.01^
**Medicaid**	3.355	0.522–6.188	0.02^
**Medicaid Managed**	2.130	1.406–2.854	**<** 0.01^
Other*	-0.184	-0.877–0.509	0.63

^Values are significant *p* < 0.05

*Self-Pay, Uninsured, Law-enforcement

**Table 4:** Binary Logistic Regression Output- Odds of Being Placed in an Inpatient Care Setting for Arthroplasty

	Odds Ratio	Confidence Interval	P-Value
**Admit Age**	1.009	1.004–1.014	< 0.01^
**Elixhauser Score**	1.025	1.017–1.033	< 0.01^

^Values are significant *p* < 0.05

**Table 5:** Odds (OR) of Being Placed in an Inpatient-Care-Setting Stratified by Insurance Status and Arthroplasty

	Hip Arthroplasty (2020 Onwards)	Knee Arthroplasty (2018 Onwards)	Ankle Arthroplasty (2021 Onwards)	Shoulder Arthroplasty (2021 Onwards)
Medicare				
OR	1.65	1.27	12.93	0.61
CI	1.01–2.70	1.01– 1.59	1.01-164.82	0.06–6.07
P-Value	0.046^	0.040^	0.049^	0.673
**Medicare Managed**				
OR	1.14	0.89	4.07	1.77
CI	0.68–1.91	0.71–1.11	0.39–42.5	0.21–14.58
P-Value	0.606	0.285	0.240	0.597
**Medicaid Managed**				
OR	1.06	0.83	—	1.63
CI	0.46–2.46	0.55–1.23	—	0.14–19.37
P-Value	0.893	0.348	—	0.697

The reference group to which these Odds Ratios correspond were privately insured patients

**^** Numbers were significant *p* < 0.05

## Results

There was a higher prevalence of patients who underwent outpatient TKA, TSA, and TAA when compared to outpatient THA (Table 2). There were significantly more patients under the age of 65 and significantly less patients over the age of 65 who underwent TAA when compared to the other forms of arthroplasty considered (Table 2). There were a significantly decreased number of Asian patients undergoing TSA and TAA, and significantly fewer Asian and “other” patients insured by Medicaid when compared to the other insurance statuses considered as shown through the Fisher’s exact test (Table 1). No other significant differences were found among patient description values in the tables above (Tables 1 and 2).

Ordinary least squares regression analysis demonstrated that both insurance status and patient age were significantly associated with Elixhauser index scores, though to different extents (Table 3). Being insured by Medicaid and Medicaid managed was strongly associated with increased Elixhauser index scores (3.355 (*p* = 0.02), 2.130 (*p* < 0.01) respectively) (Table 3). Likewise, being insured by Medicare and Medicare Managed was associated with increased Elixhauser index scores, but to a lesser degree (1.560(*p* < 0.01), 0.613(*p* < 0.01) respectively) (Table 3). Patient age was significantly, but weakly, associated (0.135, *p* < 0.01) with increased patient comorbidity status (Table 3). Patients with increased scores on the Elixhauser index were associated with slightly increased odds (OR:1.025, *p* < 0.01) of inpatient admission (Table 4). A similar trend was noted with increased patient age at admission (OR:1.009, *p* < 0.01).

Patients insured by Medicare, when compared to those privately insured, had significantly higher odds of being placed into inpatient care settings for total hip arthroplasty (OR:1.65, *p* < 0.05), total knee arthroplasty (OR:1.27, *p* < 0.05), and total ankle arthroplasty (OR:12.93, *p* < 0.05) (Table 5). Notably, this trend held true while only considering the years after which the procedure in question was removed from CMS’s inpatient only list. Patients insured by Medicare Managed, Medicaid, or Medicaid Managed did not have statistically significant odds of being placed in either care setting across the various surgical procedures considered (Table 5).

## Discussion

Studies have demonstrated the efficacy and cost-savings offered by outpatient arthroplasty procedures, advocating that similar outcomes can be produced for a margin of the cost for aptly selected patients [3, 4, 11, 13, 14]. According to our review of the literature, this is the first study that explores the association between patient characteristics, specifically insurance status, and patient placement into either inpatient or outpatient care settings for total joint arthroplasty.

With the rapid shift in the industry towards commodity outpatient surgeries such as arthroplasty, the process behind selecting ideal candidates has become more relevant now than ever before [15]. Historically, patients with pre-existing conditions such as uncontrolled hypertension or diabetes have been assigned to inpatient surgery in order to minimize risk [14]. This is reflected in many hospitals’ protocols [14, 16]. Specifically, patients treated in an outpatient center for total joint arthroplasty must be willing and able to consent, have an ASA classification less than III, must be undergoing primary arthroplasty, younger than 75, and have support at home. Inpatient criteria includes ASA classification greater than II, bleeding disorders, or poorly controlled comorbidities–specifically cardiac (heart failure), pulmonary (respiratory issues), BMI > 30, end stage renal disease [17]. Inpatient rehabilitation is also better equipped to handle the more intensive care schedules generally required by these patients [14, 18]. Although some healthcare analysts claim that physicians may be looking for ways to transition patients into outpatient care, hospitalists maintain that proper management of preexisting conditions remains the most important factor [16]. However, results from this study imply that comorbidities are not the sole determinant in choosing inpatient surgery. Medicaid and Medicaid-managed insurance statuses had the most positive association with the Elixhauser index (Table 3)—patients insured by these providers on average had the highest number of comorbidities. Yet it was Medicare insurees who had significantly higher odds of being assigned to an inpatient care setting for three of the four procedures considered. Inpatient surgery results in longer lengths of stay, fewer home rehabilitation options, and can lead to increased cost burden. In addition, increased Elixhauser index scores only led to minimally increased odds of being placed into an inpatient care-setting, further affirming these results. (Table 4).

We posit that differences in both patient preferences and reimbursement structures across various insurance statuses may partially account for this discrepancy. Recent studies have elucidated that outpatient arthroplasty could increase patient satisfaction and facilitate faster rehabilitation while minimizing dependence on hospital resources [19]. With the advent of COVID-19, special emphasis was placed on strengthening remote recovery options leading to both increased availability and efficacy [13, 20]. In some Midwestern orthopaedic clinics, up to 90% of arthroplasty patients opt for home recovery with telehealth visits, and all patients expect the option [20]. In addition, the cost savings associated with outpatient care schedules lead to lower co-insurance and deductibles for commercially insured individuals, making this a significantly more attractive choice [21].

Most patients undergoing total arthroplasty are covered under Medicare [22]. Although it might be expected that Medicare patients would share the same preferences, the differences in patient cost structures between inpatient and outpatient procedures may play a role. CMS removed arthroplasty from the inpatient-only list in order to incentivize hospitals to place more Medicare covered patients into an outpatient care setting, thereby lowering costs [11]. This is complicated by the fact that outpatient procedures are billed through Medicare part B while inpatient services are covered by Medicare part A. Unlike most private insurances and Medicare part A, Medicare part B has a 20% coinsurance rate with no cap on out-of-pocket expenses [23]. While outpatient costs may be lower as a whole, patients under Medicare part B will be responsible for 20% of the Medicare-approved amount for each service rendered [11, 24]. With increases in health advocacy and education, it is plausible that Medicare patients therefore would prefer inpatient treatment as a means to lower out-of-pocket expenses.

From a hospital perspective, inpatient procedures allow a higher rate of reimbursement at 55% compared to only 47% for the same procedure in an outpatient setting [25]. With overall reimbursement rates for all orthopaedic surgical procedures in a steady decline (1.5% per year), the potentially beneficial variations in insurance reimbursement structures may factor into hospital decision-making [11, 24, 26]. It is well-known that private insurance reimburses more for the same procedure compared to Medicare and Medicaid regardless of the care setting; however the magnitude of this difference across care settings is noteworthy [27]. A 2018 study conducted by RAND considering over $33 billion in spending from over 3000 hospitals in every state in the country concluded that, on average, private insurance reimbursed 247% of what Medicare reimbursed for the same procedure [28]. Most of this difference stemmed from the outpatient setting, where private insurance paid 267% more than Medicare when compared to only 231% more in an inpatient setting for the same service rendered [28, 29]. A systematic review of 19 studies in 2020 demonstrated an even larger discrepancy; private insurance on average paid 264% and 189% more than Medicare for the same outpatient and inpatient services respectively [27, 29]. Profit margins also differ across care settings. A 2015 report by a healthcare financial management association discovered that margins on commercial insurance reimbursement were markedly higher for outpatient care settings [15]. Medicare reimbursement margins, on the other hand, were found to be higher for inpatient services [4, 15]. With overall reimbursement rates dropping, it may make sense for hospitals to reap greater profit margins with reduced resource utilization by placing privately insured patients in an outpatient care setting. In the same vein, profits can theoretically be maximized by placing Medicare patients in an inpatient care setting. Ultimately, Medicare patients may be placed in an inpatient care setting even if eligible for outpatient surgery—likely due to patient preferences and reimbursement potential from the hospital perspective. Given that inpatient surgery leads to greater lengths of stay, increased costs to the health system, and varied rehabilitation options, it is important to understand why this trend is significant. Patient preferences held by those insured by private insurance and Medicare align well with hospital margins and reimbursement rates, a finding that warrants further research and potential policy refinement.

### Limitations and future directions

The primary limitations associated with this study stem from the sample size, the singular location utilized, inability to qualify the level of home support available for patients after discharge, and the fact that CMS only recently removed many of the procedures considered from its inpatient only list (IPO). While the sample consisted of over 6,800 cases, most of these cases (60.3%) were performed in an inpatient care setting. Additionally, most of the cases studied were hip and knee arthroplasties. The conclusions drawn by this paper primarily apply to inpatient knee and hip arthroplasty, while conclusions drawn regarding ankle and shoulder arthroplasty must be further confirmed by future works. Since ankle and shoulder arthroplasty were removed from the CMS IPO list less than two years ago, conducting a study with a similar design in the future could yield more reliable data regarding the results of this study. Certain insurance statuses, particularly Medicaid and Medicaid managed were poorly represented in the sample. Conclusions implicating these statuses must be further verified. Additionally, for analysis, return to hospital had to be used instead of readmissions to ensure that outpatient surgical candidates could also be considered—while a rather vague statistic, it was not heavily implicated in the analysis. An additional limitation is that though only primary arthroplasty was considered, there are a number of reasons why patients are subject to this form of arthroplasty, ranging in severity from elective to post-traumatic. While a majority are elective in nature, it is possible that some of the analysis may be confounded by primary arthroplasties requiring inpatient care due to their level of complexity or potential emergent status. With regards to controlling for comorbidities, the use of an index such as the Elixhauser instead of adjusting for actual diseases and conditions is an inherent limitation of this paper. Lastly, we were unable to factor the home support available to patients into the analyses conducted—the possibility that patients less likely to have access to support at home may preferentially be assigned to inpatient care settings remains unexamined. Future work should aim to gather additional variables—information regarding socioeconomic status, readmissions/returns in other local hospitals, patient satisfaction, and hospital length of stay would strengthen the conclusions of the study.

## Conclusions

Medicare patients are significantly more likely to be placed in inpatient care settings for hip, knee, and ankle arthroplasty— regardless of comorbidities or age. Medicaid patients were associated with the highest numbers of comorbidities, yet it was Medicare patients who were shown to be more likely to be placed in an inpatient setting. The fact that reimbursement rates line up relatively well with payor preferences may have unintended impacts from a practice management perspective. Future research should serve to elucidate these findings and examine their consistency across a wider population of patients nationally.

## Data Availability

The datasets used and/or analyzed during the current study are available from the corresponding author upon reasonable request.
